# Narrative Review of Pathogenetic Mechanisms and Diagnostic Biomarkers in Adipose Tissue Remodelling and Adipokine Dysregulation in Lipedema

**DOI:** 10.3390/cimb48101038

**Published:** 2026-10-07

**Authors:** Olga Sierawska, Wioleta Dudzińska

**Affiliations:** 1 Institute of Biology, University of Szczecin, 71-412 Szczecin, Poland; 2 Department of Functional Diagnostics and Physical Medicine, Pomeranian Medical University in Szczecin, 71-210 Szczecin, Poland

**Keywords:** lipedema, adipokines, adipose tissue remodelling, immunometabolism

## Abstract

**Aim:** This narrative review summarises evidence on pathogenetic mechanisms of adipose tissue remodelling and adipokine dysregulation in lipedema, focusing on adipocyte hypertrophy and hyperplasia, fibrosis, microcirculatory and lymphatic dysfunction, immune-cell alterations, and diagnostic biomarkers. **Methods:** We conducted a narrative search in PubMed, Scopus, and Google Scholar from database inception to September 2026, including publications from 2001 to 2026. Search terms included “lipedema”, “lipedema adipokines”, “lipedema biomarkers”, “adipose tissue remodelling”, “fibrosis”, “adipocyte hypertrophy”, and “adipocyte hyperplasia”, combined with specific molecules. Bibliographies were screened. This review was not registered and did not follow a systematic-review protocol. **Results:** Lipedema involves pathological subcutaneous adipose tissue remodelling, including adipocyte hypertrophy and hyperplasia, extracellular matrix fibrosis, vascular alterations, and lymphatic dysfunction. Adipose tissue macrophages are enriched in CD163+/CD206+ populations, although the M1/M2 framework inadequately represents their heterogeneity. Individual adipokines (leptin, adipocyte-type fatty acid-binding protein) show limited diagnostic value due to dependence on total adipose mass and BMI. Multicomponent signatures (adiponectin, adipocyte-type fatty acid-binding protein, fibroblast growth factor 21, chemerin, afamin, growth and differentiation factor 15) demonstrate promising performance. Additional markers include reduced tissue adipsin expression, altered endostatin, and elevated interleukin-11, interleukin-28A, and interleukin-29. Most studies are limited by small cohorts, cross-sectional designs, inconsistent controls, obesity-related confounding, and lack of external validation. **Conclusions:** Lipedema involves coordinated metabolic, inflammatory, vascular, and extracellular-matrix alterations. Single adipokines are unreliable diagnostic biomarkers. Multicomponent panels may improve differentiation from obesity, but remain exploratory. Larger multicentre studies with standardised phenotyping and external validation are needed before clinical implementation.

## 1. Introduction

Lipedema is a chronic, progressive disorder of adipose tissue (AT) characterised by abnormal accumulation of subcutaneous adipose tissue (SAT) in the limbs and affecting women almost exclusively [1]. The condition, although common and with a characteristic clinical presentation, is often wrongly diagnosed or underdiagnosed as obesity without lipedema, which in turn results in the delayed introduction of suitable management strategies [2].

As mentioned in a previous review [3], in contrast to the traditional view of AT as a passive energy reservoir, it is now recognised as a highly active endocrine organ exerting a powerful influence on the body’s metabolic, vascular, and immunological homeostasis. AT synthesises and releases a range of biologically active molecules called adipokines (e.g., leptin, adiponectin, resistin, and visfatin). Adipokines act locally and systemically to regulate metabolism and energy homeostasis, and to modulate immune responses and the course of inflammatory processes. Histopathological characterisation of lipedema adipose tissue has identified necrotising adipocytes surrounded by CD68+ macrophages forming crown-like structures [4], dilated blood and lymphatic microvessels with increased angiogenesis and macrophage enrichment [5], adipocyte heterogeneity by single-cell RNA sequencing [6], endothelial cell alterations in capillaries [7], and adipose tissue biology with weight loss effects [8]. Additional studies have examined tissue miRNAs [9], diagnostic markers in adipocyte cultures [10], and vascular changes [11,12]. In lipedema, however, pathological remodelling of the SAT in the limbs occurs, characterised by its hypertrophy, hyperplasia, local tissue hypoxia, and chronic low-grade inflammation [13]. Tissue hypoxia, while proposed as a mechanism, remains hypothetical in humans and has not been directly demonstrated.

In this context, this study aims to discuss dysregulated adipokine secretory profiles in lipedema and evaluate their potential as diagnostic and pathophysiological biomarkers. This review aims to comprehensively summarise current knowledge on adipose tissue remodelling and adipokine dysregulation in lipedema, with particular emphasis on their pathogenetic roles and potential as diagnostic biomarkers. The study focuses on characterising adipokine expression patterns in lipedema adipose tissue and serum, exploring their associations with key pathophysiological features (including adipose tissue hypertrophy, fibrosis, inflammation, and vascular dysfunction), and identifying candidates for future mechanistic studies and clinical applications. Regarding adipose tissue macrophages, the traditional M1/M2 framing is now considered outdated; human adipose tissue macrophages are more accurately described as CD163+/CD206+ enriched, consistent with lipid-associated macrophage populations [14,15].

We performed a narrative literature search in Scopus, PubMed, and Google Scholar for articles available from database inception to September 2026. The search included studies published between 2021 and 2026. The search strategy combined disease-related terms, tissue-remodelling terms, and biomarker-related terms. The main keywords were ‘lipedema’, ‘lipedema adipokines’, ‘lipedema biomarkers’, ‘adipose tissue remodelling’, ‘extracellular matrix remodelling ‘, ‘fibrosis’, ‘adipocyte hypertrophy’, ‘adipocyte hyperplasia’. These terms were also combined with the names of selected molecules, including leptin, adiponectin, chemerin, resistin, visfatin, adipsin, fibroblast growth factor 21, interleukin 11, interleukin 28A, interleukin 29, and adipocyte-type fatty acid-binding protein.

Studies were considered relevant when they investigated the clinical characteristics, histopathology, cellular composition, molecular mechanisms, adipokine profiles, inflammatory mediators, vascular or lymphatic abnormalities, or potential diagnostic biomarkers of lipedema. We considered both human studies and mechanistic laboratory studies, provided they contributed directly to the biological or diagnostic interpretation of lipedema. We examined reference lists of relevant articles and reviews to identify additional publications.

The evidence was organised thematically according to adipose tissue remodelling, fibrosis, microcirculatory and lymphatic dysfunction, stromal-vascular fraction alterations, macrophage enrichment, adipokine dysregulation, and biomarker development. Because this article provides a narrative synthesis rather than a systematic review or meta-analysis, we did not perform a formal risk-of-bias assessment, register a protocol, or quantitatively pool results. The authors interpreted the findings with particular attention to sample size, control-group selection, body mass index, disease stage, tissue depot, hormonal status, assay type, and validation procedures.

## 2. Lipedema as a Distinct Adipose Tissue Disorder

Lipedema usually presents with bilateral enlargement of the extremities, with the hands and feet relatively unaffected. Truncal or abdominal involvement may also occur, especially in more advanced cases, but its diagnostic significance is still being investigated. This distribution creates the so-called cuff sign: a distinct demarcation line above the ankles or wrists, resulting from the abrupt termination of AT deposits [16]. The cuff sign should be regarded as a supportive clinical finding, not a diagnostic test specific to the disease; the condition is usually called “painful fat syndrome” since patients report a pressing pain, a sense of heaviness, and marked tenderness in the limbs upon being touched, with the pain getting worse at the end of the day [17]. Another common symptom is increased capillary fragility, which manifests as a tendency to bruise easily, even with minimal trauma [18]. A characteristic feature of lipedema tissue is its reported resistance to traditional weight-loss methods, such as restrictive diets or intense physical activity [19]. However, the recent literature disputes this premise. While the disproportionate distribution of SAT in lipedema may persist despite weight loss, recent dietary intervention trials have shown measurable reductions in limb circumference, pain, and quality-of-life improvements with low-carbohydrate, ketogenic, and calorie-restricted diets [20,21,22]. Lifestyle interventions can improve overall metabolic health and reduce adiposity in both lipedematous and non-lipedematous compartments. However, fat loss in affected areas may be less pronounced than in unaffected regions [23,24]. Physical examination reveals joint hypermobility in nearly half of patients, as well as skin changes such as telangiectasias (spider veins) and reduced skin temperature in affected areas [25]. Although lipedema is primarily associated with the limbs, recent data indicate it can also affect the abdomen, where it shows similar characteristics of pain, fibrosis, and a lack of response to weight loss [26]. Recent histopathological and imaging studies have documented steatonecrosis and haemorrhage around immature vessels in lipedema nodules [27,28].

Several clinical classification systems describe three morphological stages of lipedema (stage 1–3), based on the severity of palpation findings in the skin and subcutaneous tissue. Some systems incorporate a fourth stage to denote lipedema with secondary lymphatic dysfunction (lipolymphedema), but this is not universally adopted [23,24]. The German S2k guideline [24] and the US standard of care [29] recognise three stages and describe lipolymphedema as a complication rather than a separate stage. In contrast, the Dutch guideline [30] and some clinical practices use a four-stage system, where stage 4 corresponds to lipolymphedema. An extended classification that incorporates intermediate stages (1.5 and 2.5) and additional features, including pain, swelling, and changes in AT distribution, was proposed [31]. Key morphological characteristics of each stage are summarised in Table 1.

Symmetrical AT accumulation is one of the most characteristic clinical features of lipedema and helps distinguish it from other conditions, such as lymphedema. This condition involves a bilateral, nearly identical increase in subcutaneous tissue volume, primarily affecting the lower extremities and, in approximately 30% of patients, the upper extremities [31]. A key feature is that AT accumulation typically stops abruptly at the ankles or wrists, creating the “cuff sign”. At the same time, the feet and hands themselves remain unchanged and slender [32].

This type of body fat distribution leads to a marked, often drastic, disproportionate body shape, characterised by massive legs or arms. At the same time, the torso remains slender, at least in the early stages of the disease [33]. The structure of accumulated fat in lipedema differs from that found in obesity; macroscopically, it is uneven, nodular, and often painful to the touch. As the disease progresses, lumps become palpable under the skin and, in advanced stages, can develop into large, deforming patches of tissue [19]. This symmetry is so characteristic of lipedema that its absence (i.e., asymmetric enlargement or swelling in only one limb) almost always suggests a different diagnosis, most often a lymphatic one. Ultimately, this specific “mirror-image” limb enlargement—which often persists despite weight loss—serves as the primary diagnostic sign prompting patients to seek help from a specialist [34].

Another integral part of the clinical picture of lipedema is persistent pain and tenderness in the AT. These symptoms are mainly localised in the front of the lower legs and thighs. Characteristically, this pain can be triggered even by gentle touch or pressure, and its intensity often increases toward the end of the day; unlike venous oedema, it does not subside with limb elevation [24,35,36].

Another significant symptom is swelling, which is typically minimal or absent in early-stage lipedema [37]. In the early stages of the disease, when present, the swelling is typically firm and non-pitting, meaning pressing the skin with a finger does not leave an indentation [38]. However, as the condition progresses, the accumulating AT may exert mechanical pressure on the lymphatic vessels, leading to secondary lymphatic drainage disorders and the development of so-called lipolymphedema in which the swelling becomes more visible and may also affect the feet and hands [39]. The European consensus and recent reviews argue that relevant oedema is usually absent in pure lipedema and that most worsening of limb circumference with disease progression is attributable to weight gain rather than fluid accumulation [24,40].

These symptoms are linked to a strong tendency to develop bruises and petechiae due to increased capillary fragility and microangiopathic changes in AT, as was shown using quantitative measurements of capillary resistance [41]. Women affected by lipedema usually find that hematomas appear following very slight or even completely unnoticed mechanical trauma [42]. The chronic pain, the disproportionate limb enlargement which may persist despite weight-loss efforts, and the tendency to bruise all make it hard for patients to carry out their everyday physical activities and greatly reduce their quality of life, often leading to serious psychological stress and anxiety [43].

Distinguishing lipedema from obesity and various forms of lipodystrophy is crucial for accurate diagnosis, as these conditions—despite certain visual similarities—differ in aetiology, clinical course, and metabolic profile. The main difference between lipedema and obesity lies in body proportions and responses to weight-loss attempts. In obesity, AT accumulation is typically proportional and involves the trunk, whereas in lipedema, the limbs enlarge symmetrically and in isolation, with the trunk remaining relatively slender [44]. The disproportionate distribution of SAT in lipedema may persist despite weight loss, although lifestyle interventions can still improve overall metabolic health and reduce adiposity in non-lipedematous compartments [23]. In addition, lipedema is accompanied by spontaneous pain and tenderness, which are rare in obesity without lipedema, as well as a tendency to bruise due to vascular fragility [33]. Patients with lipedema may also have a normal BMI (18.5–24.9 kg/m^2^), whereas obesity is defined as BMI ≥ 30 kg/m^2^ [45].

The causes of lipedema are complex and multifaceted. Although the exact pathophysiology is not yet fully understood, several mechanisms have been proposed, including genetic, hormonal, and vascular factors, as well as alterations in connective tissue structure. Heredity plays a significant role in lipedema development, and a family history is reported in 30% to 89% of patients [16]. Analyses suggest an autosomal dominant inheritance pattern with incomplete penetrance. Candidate genes and mutations have been proposed based on single-family reports and small cohorts, but large-scale studies have not confirmed a monogenic cause. For example, a mutation in the *AKR1C1* gene, which is responsible for progesterone metabolism, was reported in a single family and may lead to excessive SAT accumulation [46]. Other studies link lipedema to mutations in genes such as *PIT-1* (affecting pituitary hormones) [47]; *NSD1* (encoding a histone methyltransferase enzyme); and *MTHFR* polymorphisms [16,47]. However, genome-wide association studies (GWAS) found no monogenic cause, suggesting lipedema is likely a polygenic disorder influenced by multiple genetic and environmental factors [48,49].

The disease affects almost exclusively women and typically manifests or worsens during periods of rapid hormonal changes, such as puberty, pregnancy, or menopause, suggesting an oooestrogen-related aetiology, as estrogen influences the expression of genes that regulate the endothelial barrier and the sympathetic innervation of AT. These patients also show changes in the distribution of estrogen receptors (alpha and beta) in AT [42]. Vascular dysfunction, including dilated blood and lymphatic microvessels and increased angiogenesis, has been documented in lipedema adipose tissue [5,7]. Adipose tissue macrophages in lipedema show CD163+/CD206+ enrichment rather than classical M1/M2 polarisation, consistent with lipid-associated macrophage phenotypes described in obesity [14,15].

## 3. Adipose Tissue Remodelling in Lipedema

### 3.1. Adipocytes Hypertrophy and Hyperplasia

Lipedema is characterised by pathological, bilateral, and symmetrical accumulation of AT, resulting from two parallel cellular processes: hypertrophy, which is the enlargement of adipocytes, and hyperplasia, defined as an increase in the number of adipocytes [50].

In lipedema, mature adipocytes enlarge significantly, particularly in affected areas (e.g., the thighs), compared to healthy areas such as the abdomen. This hypertrophy appears stage-dependent, with the most pronounced increase in cell size at stage 2, while differences at stage 1 are minimal and those at stage 3 are smaller and likely confounded by BMI. These findings are consistent with histopathological observations of adipocyte hypertrophy and crown-like structures in lipedema tissue [4,5]. Excessively enlarged adipocytes can compress capillaries, leading to local tissue hypoxia, which triggers inflammation, macrophage recruitment, and fibrosis [50]. Tissue hypoxia, while proposed as a mechanism linking hypertrophy to inflammation, remains hypothetical in humans and has not been directly demonstrated in lipedema.

Lipedema is also associated with excessive proliferation of adipocyte precursors. This process, known as hyperplasia, is driven by cell cycle abnormalities and increased expression of proliferation-related genes, such as *BUB1*, *CCND1*, and *CDC20* [51,52]. ZNF423 also plays a key role as the dominant regulator of early adipocyte differentiation; elevated levels promote a hyperplastic tissue phenotype [53]. As the disease progresses, growing adipocytes impair lymphatic drainage and accumulate interstitial fluid, which supplies nutrients and further stimulates pathological adipocyte expansion [54]. Recent single-cell RNA sequencing analyses have revealed adipocyte heterogeneity in lipedema, providing molecular insights into hyperplastic and hypertrophic subpopulations [6].

Distinguishing between hypertrophy and hyperplasia is crucial for treatment. While standard weight-loss methods (such as diet, exercise, and bariatric surgery) can reduce hypertrophic cells to some extent, hyperplastic cells respond only modestly to these interventions [55]. Lymph-sparing liposuction or dermato-fibro-lipectomy procedures can permanently reduce adipocyte numbers in affected areas and improve symptoms [56,57].

### 3.2. Fibrosis

Progressive interstitial fibrosis is a key component of lipedema pathophysiology and directly affects SAT structure and disease course [38]. AT undergoes profound remodelling and increased stiffness, as shown by thickening of interlobular septa and changes in extracellular matrix composition—both associated with the loss of skin elasticity and the appearance of hard, palpable nodules and fibrotic bands. On clinical examination, these fibrotic changes present as subcutaneous nodules that may initially feel like small grains of rice or pearls and, with disease progression, enlarge into walnut-sized or larger lobules of tissue, deforming the skin [23,58]. This phenomenon accounts for the distinctive, hard, and uneven texture of lipedema tissue, frequently described as the mattress sign or orange peel skin. It causes chronic pain, tenderness upon pressure, and a constant feeling of heaviness in the limbs [13].

Clinical studies indicate that lipedema fibrosis is closely related to disease stage and primarily affects the lower extremities. Importantly, from a diagnostic standpoint, pathological fibrosis is significantly elevated as early as stage I, meaning it develops very early—often before marked adipocyte hypertrophy [42]. This suggests that ECM remodelling may precede metabolic changes in adipocytes and could serve as an early marker, though this requires validation in diagnostic studies. Transcriptomic analyses have identified upregulation of ECM-related genes (e.g., *LUM*, *EMILIN2*, *FBN1*) in stage II and III lipedema, indicating active ECM remodelling and proteoglycan–microfibril scaffold degradation at the molecular level [59]. Histopathological studies also show increased collagen deposition (fibrosis) in affected subcutaneous adipose tissue compared with unaffected areas and lean controls, even in early disease stages (1–2) [7]. These findings suggest that ECM alterations occur at both the transcriptional and tissue levels and may contribute to the mechanical stress, inflammation, and pain characteristic of lipedema. Some researchers hypothesise the exact opposite relationship, in which a dense, fibrotic ECM physically limits further adipocyte proliferation in the early stages of the disease [38,60]. As the disease progresses to subsequent stages, the fibrotic process intensifies and correlates with the severity of symptoms [13,61].

**Figure 1:**
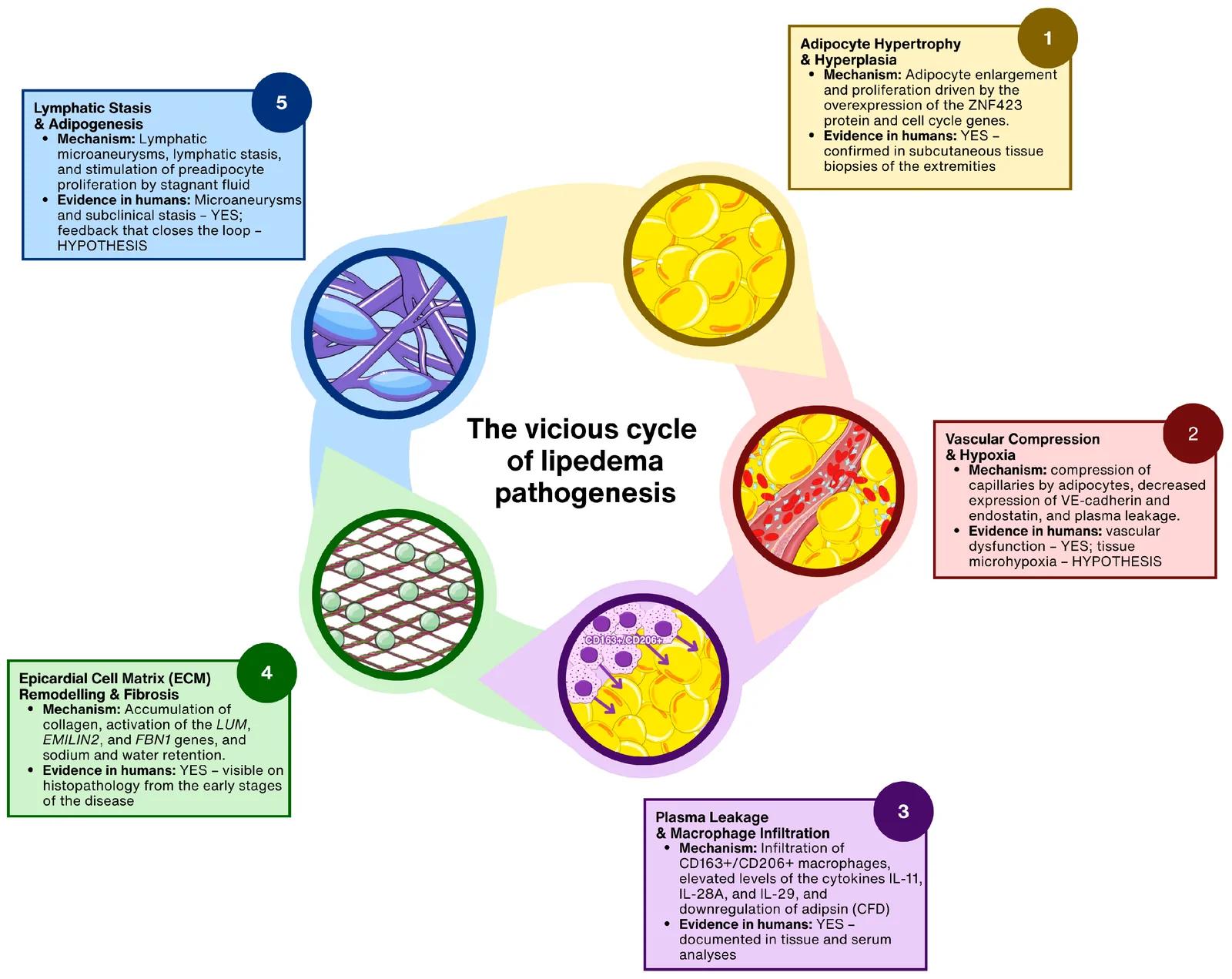
Schematic representation of the vicious cycle in lipedema pathogenesis. Five interconnected stages: (1) adipocyte hypertrophy and hyperplasia in subcutaneous adipose tissue of affected limbs; (2) mechanical compression of capillaries by enlarged adipocytes leading to reduced blood flow and local tissue hypoxia (this step remains hypothetical in humans and lacks direct evidence); (3) increased capillary permeability with plasma protein extravasation and recruitment of CD163+/CD206+ macrophages (traditionally described as M2-like phenotype); (4) fibroblast activation with excessive collagen deposition (particularly type VI) and glycosaminoglycan-mediated fluid/sodium retention; (5) lymphatic vessel compression with microaneurysm formation, protein-rich fluid stasis, and adipogenesis stimulation that closes the pathophysiological loop. Image adapted from Servier Medical Art (https://smart.servier.com/), licenced under CC BY 4.0 (https://creativecommons.org/licences/by/4.0/).

The pathogenesis is based on a “vicious cycle” in which microcirculatory disorders and chronic inflammation play key roles: adipocyte hypertrophy and interstitial fluid accumulation mechanically compress capillaries, causing local hypoxia. This “vicious cycle” model, while widely cited, is based primarily on indirect evidence and one review; direct human data supporting each step are limited to histopathological observations [4,5,7] and small ultrasound/histology series showing steatonecrosis and haemorrhage around immature vessels [27,28]. Hypoxia causes endothelial damage, increased vascular permeability, and partial adipocyte necrosis, which stimulates macrophage recruitment, particularly pro-inflammatory M1 macrophages [62]. The M1/M2 classification is now considered outdated for adipose tissue macrophages; findings are better described as CD163+/CD206+ enrichment, with stage-dependent shifts in macrophage phenotype [14,15]. Chronic, low-grade inflammation strongly stimulates fibroblasts to synthesise collagen. At the same time, glycosaminoglycan (e.g., hyaluronic acid) composition in the ECM changes; these molecules strongly bind water and sodium, increasing fluid retention and impairing lymphatic drainage. In advanced cases (lipolymphedema), fibrotic AT deposits can mechanically block lymphatic vessels, leading to secondary lymphedema [63]. Figure 1 schematically illustrates the key steps in this pathophysiological cycle.

Anatomically, classic fibrosis associated with lipedema primarily affects the extremities, whereas the abdominal subcutaneous tissue is typically normal. However, this changes in abdominal lipedema, which typically develops in the advanced stages of the disease, with progression of subcutaneous tissue involvement in the trunk [64]. In such cases, macroscopic and ultrasonographic examinations reveal abundant, dense fibrous tissue and thickened, irregular connective tissue septa located in the subcutaneous tissue, predominantly in the superficial layer, with possible involvement of the deep layer in advanced cases [26].

### 3.3. Microcirculatory and Lymphatic Disorders

Microcirculatory disorders and lymphatic dysfunction are key pathophysiological bases of lipedema [65]. As the disease progresses, progressive adipocyte hypertrophy exerts physical pressure on the capillaries, leading to local tissue hypoxia. However, direct evidence of tissue hypoxia in human lipedema is lacking, and this remains a proposed mechanism rather than a fact. Hypoxia becomes a powerful inducer of pathological angiogenesis and directly causes endothelial barrier dysfunction [66]. At the molecular level, damage to intercellular junctions in blood vessels may occur, as shown by reduced expression of proteins that maintain vascular barrier tightness and integrity, including VE-cadherin (*CDH5*) [53]. However, these findings require further validation in the context of lipedema. This damage significantly increases capillary permeability, causing proteins and fluids to leak into the surrounding interstitial space (extravasation). Microcirculatory dysfunction and disturbances in pressure regulation within the vascular bed further exacerbate the situation, promoting additional fluid leakage [67]. Vascular alterations in lipedema include dilated blood and lymphatic microvessels, increased angiogenesis, and endothelial cell changes, as demonstrated in previous studies [5,7]. At the same time, increased extracellular matrix pressure causes lymphatic vessels in advanced cases to lose their ability to transport fluid effectively, resulting in fluid stagnation and potentially leading to secondary lymphatic insufficiency [68].

In the early stages of the disease, the lymphatic system attempts to compensate by increasing drainage of excess interstitial fluid. However, as the disease progresses, fluid production significantly exceeds the lymphatic system’s transport capacity. Chronic stasis of protein-rich fluid causes inflammation at the site and, in turn, stimulates extracellular matrix fibrosis [67]. Under the microscope, lymphatic vessels show subclinical lymph stasis (lymphostasis), a twisted course, and characteristic microaneurysms that give the vessels a “bead-like” appearance (microlymphatic aneurysms) [69]. Imaging studies confirm markedly slowed lymph flow, further exacerbated by excessive sodium accumulation in the tissue—elevated sodium concentrations bind to proteoglycans and directly impede lymph drainage [70]. Ultimately, mechanical compression from expanding AT and extensive fibrosis compresses and blocks the lymphatic vessels. This results in secondary damage to the lymphatic system and the development of so-called lipolymphedema (in the advanced stage), in which lymphedema joins the symptoms of lipedema [71]. Crucially, chronic lymphatic stasis and accompanying inflammatory factors may promote adipogenesis by stimulating further adipocyte proliferation and differentiation, ultimately closing and driving the destructive pathophysiological cycle of lipedema [72,73].

### 3.4. Changes in the Cells of the Vascular Stroma

The stromal vascular fraction (SVF), which includes stem cells and other stromal and perivascular cells, appears to exhibit several significant abnormalities in lipedema. In a previous study [74], diseased adipose tissue yielded a higher total number of SVF cells, and flow cytometric analysis showed higher percentages of the CD90+ (mesenchymal) and CD146+ (endothelial–pericytic) populations than in the control group. However, the control group included only 3 individuals compared with 20 patients with lipedema, and the groups differed by about 6 units in mean BMI, which significantly limits interpretation. A later study [53], conducted on a larger and better-matched cohort, did not confirm differences in the percentage of CD146+ cells between the lipedema group and the control group; the authors also suggest that earlier findings regarding CD146 may have been partly an artefact related to the presence of CD45+ cells, and the CD90 marker was not assessed in their study. Despite these discrepancies, the elevated expression of CD90 and CD146 observed in the study is sometimes interpreted as a potential indicator of chronic capillary damage, which increases the need for tissue repair and protection. At the molecular level, CD90 activates the AKT kinase pathway and drives overexpression of cyclin D1 (CCND1) [75], which may promote cell proliferation and contribute to the hyperplasia typical of lipedema. CD146, on the other hand, acts as a receptor for the ANGPTL2 protein and is implicated in promoting adipogenesis and lipogenesis; in lipedema, this may contribute to abnormal tissue remodelling, although data on the adipogenic potential of SVF are inconclusive [53,76]. These changes within SVF cells are associated with microangiopathy and may indirectly promote ECM fibrosis, which disrupts tissue homeostasis and leads to abnormal connective tissue remodelling [53,59].

Another key aspect of vascular stroma pathology in lipedema involves metabolic and secretory changes. In SVF cells from patients with lipedema, ZNF423 is significantly overexpressed; it is a major regulator of early adipocyte differentiation [53]. Interestingly, an assessment of mitochondrial function in SVF cells revealed a significantly higher maximal oxygen consumption rate (OCR) than in the control group, indicating increased mitochondrial respiratory capacity, although basal OCR and its ATP-linked and non-mitochondrial components did not differ; these findings come from a small cohort of 4 controls vs. 5 lipedema patients [77]. Separate studies; however, have shown increased infiltration of M2-phenotype macrophages in lipedema SVF, evidenced by elevated CD163 expression at both the mRNA and protein/cytometry levels [52,78]. The M2-phenotype terminology is imprecise; these findings are more accurately described as CD163+/CD206+ macrophage enrichment, consistent with lipid-associated macrophage populations [14,15]. Although a direct link between OCR and M2 macrophage abundance has not been established, the coexistence of heightened oxidative metabolism and an M2-skewed immune milieu suggests that macrophages may contribute to the metabolic reprogramming of the stromal compartment in lipedema. At the secretory level, the conditioned medium derived from SVF cells of patients with lipedema shows a significant, selective decrease in the level of the cytokine IL-8. At the same time, this medium has a destructive effect on healthy vascular endothelial cells—under its influence, intercellular junctions are weakened, including through reduced expression of VE-cadherin, which significantly increases capillary permeability and promotes fluid leakage into the intercellular space; this mechanism is multifactorial and does not depend solely on IL-8 [53].

Cells of the vascular stroma fraction are also a source of abnormalities in vesicular communication. Analyses of small extracellular vesicles (sEVs) released by lipedemic SVF cells revealed a unique and altered microRNA profile. Significant differences were demonstrated, among other things, in the expression of key miRNAs, such as miR-16-5p, miR-24-3p, miR-29a-3p, miR-130a-3p, miR-454-3p, miR-144-5p, and let-7c-5p; in sEVs from the SVF of patients with lipedema, four of these (miR-16-5p, miR-24-3p, miR-29a-3p, miR-454-3p) were downregulated, while three (miR-144-5p, miR-130a-3p, let-7c-5p) are elevated compared to the control group. Further study [9] explored tissue miRNA profiling in adipose disorders, although lipedema-specific data remain limited. For example, reduced miR-16-5p levels may disrupt cell cycle control (e.g., by regulating cyclins, including CCND1) and promote increased adipocyte proliferation [79]. All these changes confirm that the cells of the vascular stroma in lipedema are not merely a passive background for adipocytes, but constitute an active, pathologically altered environment that, through disturbances in angiogenesis, secretion, and proliferation, directly initiates and sustains the clinical phenotype of the disease; the functional consequences of individual miRNAs are partly hypothetical and require further study.

### 3.5. Predominance of CD163+/CD206+ Macrophage Enrichment

A distinctive feature of the inflammatory profile of adipose tissue in lipedema, particularly in early stages, is the increased proportion of macrophages expressing M2-associated markers such as CD163 and MRC1/CD206. The M1/M2 framework is now considered outdated for adipose tissue macrophages; findings should be described as CD163+/CD206+ enrichment, reflecting lipid-associated macrophage phenotypes rather than classical polarisation states [14,15]. While classically associated with anti-inflammatory functions, the functional significance of this M2-like profile in lipedema and its stage-dependent changes requires further investigation [80]. This predominance of M2 cells over pro-inflammatory M1 macrophages is most evident in the early stages of the disease, as evidenced by a significantly elevated M2-to-M1 ratio in the subcutaneous tissue of the thighs in stage 1 patients. However, as the disease progresses to stage 3, this protective, anti-inflammatory environment weakens in a stage-dependent manner, and the M2:M1 ratio decreases in favour of pro-inflammatory macrophages [50]. These stage-dependent shifts in CD163+/CD206+ macrophage enrichment require validation in larger cohorts with single-cell resolution. Although M2-like macrophages perform immunomodulatory functions, their chronic presence may contribute to tissue remodelling. In other contexts, M2 macrophages have been implicated in pathological angiogenesis and fibroblast activation, suggesting a potential role in lipedema fibrosis that requires direct validation.

Furthermore, recent studies indicate that in lipedema, M2 macrophages may adopt a distinct immunosuppressive state that can modulate adipocyte progenitor behaviour and differentiation, thereby directly affecting the tissue’s metabolic homeostasis [81]. In particular, it was reported [78] that a CD163^+^/CD206^+^ M2-like macrophage infiltrate in lipedema adipose tissue, and that, in co-culture models, repolarisation of these macrophages normalises adipogenic differentiation of adipose-derived stem cells, supporting a causal role for M2 cells in regulating adipogenesis in this condition. These findings do not necessarily contradict earlier proposals that M2 macrophages can promote adipogenesis [80], as the net effect likely depends on macrophage activation state, disease stage, and the specific readout (progenitor proliferation vs. lipid accumulation in differentiating adipocytes).

However, the functional consequences of macrophage-adipocyte crosstalk in lipedema remain incompletely understood and warrant further mechanistic studies.

## 4. Adipokines in Lipedema

### 4.1. Leptin

Leptin is a major adipokine, and its serum concentration strongly correlates with total body fat mass and BMI. It plays an important role in regulating energy balance by acting on satiety centres in the hypothalamus, thereby controlling food intake and energy expenditure. In addition, leptin exhibits pro-inflammatory, profibrotic, and proangiogenic effects, influencing immune function, adipose tissue metabolism, and vascular homeostasis [82]. Studies on the role of leptin in lipedema pathogenesis show marked discrepancies, depending on whether researchers analysed circulating hormone levels, local gene expression in tissue, or cell behaviour in in vitro cultures. Regarding serum leptin concentrations, studies found [52,83] no significant differences between patients with lipedema and their BMI-matched control group. In turn, it was observed [84] that in female patients with lipedema (stages 1–3), serum leptin levels were generally elevated, and in stage 1 there was a marked disruption of the physiological, positive correlation between leptin levels and BMI; these results may be partially modulated by AT mass and coexisting obesity. Conflicting data also exist regarding leptin (*LEP*) gene expression in thigh AT: it was reported [52] that there were no changes in its expression at the tissue level, whereas it was demonstrated [50] a significant reduction in *LEP* expression in thigh SAT biopsies from patients in advanced stages (2 and 3). Similar contrasts are evident in in vitro cell culture studies. Increased leptin gene expression was detected in adipocytes differentiated from thigh adipose-derived stem cells (ASCs-T) from patients with lipedema [85], whereas another study [86] showed that these cells, during in vitro culture, release significantly lower amounts of leptin protein into the supernatant compared to cells from healthy individuals; this discrepancy may result from differences in protocols and the degree of adipocyte maturity, as mRNA expression does not always translate proportionally into protein secretion.

### 4.2. Adiponectin

Adiponectin is one of the major adipokines secreted almost exclusively by adipocytes; its serum concentration is strongly negatively correlated with body fat mass and BMI. Adiponectin plays an important metabolic role—it increases tissue sensitivity to insulin, stimulates fatty acid oxidation in muscle, and inhibits gluconeogenesis in the liver. It also shows strong anti-inflammatory, anti-fibrotic, and cardioprotective properties by inhibiting macrophage activation, fibroblast proliferation, and endothelial damage [87]. As with leptin, adiponectin analysis in lipedema reveals significant discrepancies between studies of circulating levels and in vitro analyses of local tissues. Studies of blood adiponectin concentrations predominantly show elevated levels in patients with lipedema compared with obese women without the condition. It was reported [83] that plasma adiponectin concentrations were significantly higher in patients with lipedema than in obese women in the control group. Similarly, it was reported [88] that women with lipedema had significantly higher serum adiponectin concentrations than obese patients without the condition, which the authors interpret as a potential indicator of a metabolically healthier expansion of SAT. Interestingly, it was observed [84] that in the early phase of the disease (stage 1), the physiological negative correlation between adiponectin levels and BMI is markedly disrupted and becomes significantly weakened. At the molecular level, however, it was found [52] that there were no differences in adiponectin gene (*ADIPOQ)* expression in SAT in patients with lipedema compared to controls; similar results (no significant differences) were observed in 3D models. In in vitro cell culture results are again inconsistent. In a previous study [86], stem cells from patients with lipedema secreted significantly less adiponectin into the culture supernatant following adipogenic stimulation. A completely different trend was reported [85]; in differentiated adipocytes, they observed a statistically non-significant upward trend in *ADIPOQ* gene expression, which the authors attributed to high sample variability; this discrepancy may result from differences in protocols and the degree of adipocyte maturity, as mRNA expression does not always translate proportionally into protein secretion.

### 4.3. FGF21

Analyses of fibroblast growth factor 21 (FGF21) levels in lipedema studies yield highly variable—and, in some respect, even contradictory—findings that depend on the clinical phenotype under study and the selection of the comparison group. In a previous study [88], serum FGF21 concentrations were significantly lower in women with lipedema than in obese patients without the condition, making this parameter an independent predictor of a lipedema diagnosis. A one-standard-deviation increase in FGF21 concentration was associated with a 79% reduction in the likelihood of diagnosis; after excluding healthy individuals from the analysis, this increased to 89.7%. The authors suggest that lower FGF21 levels in the classic course of lipedema may reflect a metabolically healthier SAT phenotype, which, unlike visceral obesity, is not subjected to such severe metabolic and inflammatory stress. In contrast, a comprehensive plasma proteomic profiling study [83] found no statistically significant differences in circulating FGF21 levels between patients with lipedema and an age- and BMI-matched control group.

### 4.4. IL-11

Interleukin 11 (IL-11) is another cytokine worth examining in lipedema. In a previous study [77], patients with lipedema had significantly elevated serum IL-11 levels (2.64 ± 0.65 pg/mL) compared with a BMI-matched control group (2.07 ± 0.51 pg/mL). From a pathophysiological perspective, this cytokine is an important regulator of adipogenesis and adipose-derived stem cell (ADSC) proliferation; it binds to the IL-11Rα receptor and modulates pathways affecting cell differentiation and survival, with its action being context-dependent [89]. The main source of IL-11 in adipose tissue is the stromal-vascular fraction (SVF), whose abundance is significantly increased in the pathologically altered subcutaneous tissue of patients with lipedema. Researchers suggest that elevated levels of circulating IL-11, along with changes in the levels of other specific cytokines (such as IL-28A and IL-29), may contribute to the characteristic immunological niche and local inflammation associated with lipedema, which distinguishes it from classic obesity; however, it is important to note that serum IL-11 measurements (multiplex) are sensitive to sampling conditions and the type of assay.

### 4.5. Chemerin

Chemerin is another important adipokine that regulates adipogenesis, angiogenesis, and immune cell recruitment; its analysis provides valuable data for the differential diagnosis of lipedema. In a previous study [88], serum chemerin concentrations differed significantly by patient phenotype. The median concentration in patients with lipedema without obesity was 166.6 ng/mL; in women with lipedema and coexisting obesity, it increased to 194.2 ng/mL and was highest in the group with obesity but without lipedema (236.0 ng/mL). In contrast, in healthy, lean individuals in the control group, it was 158.4 ng/mL (*p* < 0.001). Multivariate analyses (PLS-DA) confirmed the high diagnostic value of chemerin (VIP score > 1), positioning it—alongside FGF21—as an independent predictor that significantly reduces the risk of a lipedema diagnosis (after adjusting for age, body fat percentage, HbA1c, and hsCRP). A one-standard-deviation increase in chemerin concentration was associated with a 56% reduction in the likelihood of a lipedema diagnosis across the entire cohort; after excluding healthy individuals, this reduction reached 66.5%. From a pathophysiological perspective, the authors suggest that the trend toward lower chemerin concentrations in patients with lipedema may indicate that SAT expansion in this condition is associated with less hypoxia and milder, low-grade inflammation than in simple obesity. In contrast, in a previous study in premenopausal women, serum chemerin concentrations did not differ significantly between patients with lipedema (126 ± 39 ng/mL) and BMI-matched obese women in the control group (141 ± 39 ng/mL) [11]. However, chemerin levels positively correlated with BMI in the control group. In contrast, in patients with lipedema, this correlation was near statistical significance, accompanied by a strong positive correlation between chemerin levels and total fat mass. Additionally, in healthy women, chemerin levels showed a significant positive correlation with endostatin (an angiogenesis inhibitor), whereas this was not the case in patients with lipedema.

### 4.6. AFABP

Adipocyte-type fatty acid-binding protein (FABP4/AFABP) plays a significant role in the differential diagnosis of lipedema, demonstrating value as a systemic biomarker while remaining stable at the level of local gene expression. In a previous study [88] evaluating the circulating serum adipokine profile, it was shown that AFABP concentrations differed significantly depending on the patients’ phenotype (*p* < 0.001): the median concentration in patients with lipedema without obesity was 21.5 µg/L; in women with lipedema and coexisting obesity, it rose to 37.1 µg/L, while the highest values were observed in the group with obesity without lipedema (43.7 µg/L). Multivariate analyses (PLS-DA) confirmed the high diagnostic value of AFABP (VIP score > 1), positioning this protein as a valuable component of the adipokine signature. However, in logistic regression, chemerin and FGF21 were the independent predictors of lipedema, whereas AFABP primarily reflected total AT mass.

However, a completely different profile is observed at the molecular level of adipose tissue. In a previous study [53], gene expression analysis revealed no statistically significant differences in *FABP4* mRNA levels in SAT (thighs and abdomen), the stromal–vascular fraction (SVF), isolated endothelial cells (EC), pericytes (PC), and cultured adipose-derived stem cells (ASC P2) between the control group and patients with lipedema. The absence of changes in local transcription of this gene is also supported by a previous study [50], in which *FABP4* expression in SAT biopsies from the thighs was stable and did not differ significantly between patients with stages 2–3 lipedema and healthy women in the control group. These results suggest that while circulating serum AFABP concentrations strongly reflect systemic adipose tissue mass and associated metabolic disorders, local FABP4 gene expression in vascular niches and progenitor cells of the thighs remains unchanged throughout lipedema pathogenesis.

### 4.7. Adipsin

Adipsin (Complement Factor D, CFD), an adipokine with serine protease properties and a key component of the alternative complement activation pathway, exhibits significant expression abnormalities in the adipose tissue of patients with lipedema. In a previous genetic profiling study [52], local expression of the adipsin gene (CFD) in SAT was significantly reduced in women with lipedema compared with a control group with matched BMI. This local transcriptional silencing at the tissue level contrasts with the expected biology of the adipsin-acylation stimulating protein (ASP) pathway: adipsin generates C3a and its metabolite ASP, which stimulate triglyceride synthesis and fatty acid storage in adipocytes, suggesting that reduced adipsin would theoretically limit rather than promote fat expansion [90,91]. A proposed hypothesis [92] posits that dysregulation of the endotoxin–complement cascade—including altered adipsin activity—may contribute to chronic low-grade inflammation and pathological adipose tissue expansion in lipedema, although the precise mechanism remains unclear. In a previous study [84], proteomic analyses revealed that in patients with lipedema, the protein signature associated with activation of the complement system and coagulation cascades in adipose tissue is reduced. Together, these findings suggest that local attenuation of complement activity may contribute to the pathological expansion and cellular abnormalities of SAT in lipedema; available data primarily concern tissue expression and tissue signatures, with no clear evidence of decreased serum CFD.

### 4.8. LCN2

Lipocalin-2 (LCN2), a recognised marker of inflammation associated in the literature with obesity, insulin resistance, and hyperglycemia, shows no significant systemic changes in studies of lipedema, allowing a clear distinction between this condition and classic metabolic obesity. In a previous study [52], researchers measured serum concentrations of this adipokine in female patients after an overnight fast. They found no significant difference between women with lipedema and the BMI-matched control group. A similar conclusion is supported by a previous study [88], which assessed serum lipocalin-2 concentrations in women divided into four clinical groups (lipedema, lipedema with obesity/overweight, obesity/overweight alone, and healthy controls). No statistically significant differences were found between the groups (*p* = 0.558), indicating that circulating lipocalin-2 levels remain stable in lipedema, regardless of the severity of tissue changes or obesity. The absence of elevated LCN2 levels in lipedema contrasts with findings in metabolic obesity and suggests that lipedema does not exhibit the classic systemic inflammatory profile associated with insulin resistance. However, the absence of elevation of a single marker does not rule out other forms of inflammation.

### 4.9. Afamin, GDF15, Progranulin

Data on afamin, growth and differentiation factor 15 (GDF15), and progranulin (PGRN) come from a single previous publication [88]. In this study, which evaluated the profile of thirteen circulating adipokines in 79 women, the authors measured afamin, GDF15, and PGRN concentrations in the serum of patients with lipedema alone, lipedema with coexisting obesity, obesity alone without lipedema, and healthy controls. Direct one-way comparisons of these three proteins showed no statistically significant differences between groups. The median serum afamin concentration was 63.4 mg/L in lipedema, 72.0 mg/L in lipedema with obesity, 75.2 mg/L in obesity alone, and 67.3 mg/L in healthy women (*p* = 0.075). For GDF15, these medians were 447.6 ng/L (lipedema), 490.7 ng/L (lipedema and obesity), 535.4 ng/L (obesity), and 580.8 ng/L (control; *p* = 0.250), while for progranulin, these values were 35.9 µg/L, 34.6 µg/L, 39.0 µg/L, and 35.9 µg/L, respectively (*p* = 0.091). Despite the absence of direct quantitative differences in serum, advanced multivariate analysis (PLS-DA) assigned afamin and GDF15 a high classification value (VIP score > 1), which positions them—alongside adiponectin, AFABP, FGF21, and chemerin—as key components of the adipokine signature that facilitate the differentiation of patients with lipedema from those with simple obesity; however, in logistic regression, chemerin and FGF21 were independent predictors of lipedema. In contrast, progranulin showed no diagnostic value in multivariate models (VIP score < 1), suggesting that its systemic levels do not reflect the specificity of the lipedema pathomechanism.

### 4.10. Resistin and Visfatin

Studies on serum concentrations of resistin (RETN) and visfatin (VISF) in patients with lipedema provide evidence that global systemic abnormalities are absent. In a previous study [93], which assessed the adipokine profile in 19 patients with lipedema (mean BMI 34.4) and 15 healthy women (mean BMI 25.5), the authors found no statistically significant differences in circulating levels of either adipokine (*p* > 0.05). The mean resistin concentration was 304.1 ± 186.8 pg/mL in patients with lipedema compared to 392.0 ± 369.3 pg/mL in controls (*p* = 0.84), while VISF concentrations were nearly identical in both groups (0.16 ± 0.04 ng/mL vs. 0.16 ± 0.05 ng/mL, *p* = 0.78). These findings suggest that, unlike in metabolic obesity, circulating RETN and VISF levels are not elevated in lipedema, although the substantial BMI difference between groups (34.4 vs. 25.5) limits direct comparisons. Correlation analyses revealed no significant associations between RETN or VISF levels and ultrasound-measured skin or subcutaneous tissue thickness in patients with lipedema. The ultrasound thickness measurements in this cohort can be interpreted in the context of proposed diagnostic cut-offs for lipedema [57], which define threshold values for the pre-tibial region (>11.7 mm), anterior thigh (>17.9 mm), and lateral leg (>8.4 mm) to distinguish lipedema from non-lipedema tissue [94]. The absence of significant correlations, despite numerical trends in opposite directions, suggests that the unique pathophysiology of subcutaneous adipose tissue in patients with lipedema may influence local adipokine secretion patterns differently from those in physiological adipose tissue; Table 2 summarises selected adipokines as potential lipedema biomarkers.

## 5. Biomarker Potential

The diagnostic potential of adipokines and other circulating cytokines and regulators in the serum or plasma of patients with lipedema is gaining importance as a potential objective tool that could improve the differential diagnosis of this condition from simple obesity. Until now, this distinction has relied almost exclusively on subjective clinical examination, although objective adjunct tools have been developed to support diagnosis. Dual-energy X-ray absorptiometry (DXA) leg-to-total fat ratios [95], ultrasound thickness criteria [94], and validated screening questionnaires [96] have been proposed as complementary diagnostic aids. A research case definition has also been proposed to standardise participant selection in studies [97]. Because the concentrations of individual classical markers, such as leptin or AFABP, depend strongly on total body fat content and correlate with BMI, single serum measurements rarely show sufficient discriminatory power in studies involving weight-matched control groups. However, recent studies suggest that multicomponent biomarker signatures may offer improved discriminatory performance.

In a previous study [88], advanced multivariate analysis (PLS-DA) identified six variables with VIP scores above 1: adiponectin, AFABP, FGF21, chemerin, afamin, and GDF15. Notably, afamin and GDF15 showed discordant results between univariate and multivariate analyses: while individual group comparisons did not reach statistical significance, both variables contributed substantially to the PLS-DA model (VIP > 1). This discrepancy likely reflects multivariate methods’ ability to capture covariance patterns and interactions between variables not apparent in univariate testing. Still, it also raises the possibility of overfitting in small cohorts. For FGF21, each standard deviation increase was associated with an odds ratio of 0.21 (95% CI 0.05 to 0.58) for lipedema diagnosis, corresponding to a 79% reduction in odds; for chemerin, the odds ratio was 0.44 (95% CI 0.19 to 0.89), corresponding to a 56% reduction in odds. An integrated decision-tree model combining the concentrations of chemerin, FGF21, and adiponectin differentiated participants with lipedema from women with obesity without lipedema with 85% accuracy, 93% sensitivity, and 75% specificity in a subset excluding lean controls (*n* = 69); in the full cohort, the model achieved 74.7% accuracy and 52.6% specificity, and no cross-validation was performed. These findings should be interpreted as exploratory multivariate classification rather than independently validated diagnostic biomarkers; the absence of cross-validation and the limited sample size increase the risk of overfitting, underscoring the need for external validation in independent, multicentre cohorts before clinical implementation. Endostatin (a potent inhibitor of angiogenesis) also appears to be a valuable vascular marker; its serum concentration in premenopausal patients with lipedema is significantly reduced (85 ng/mL vs. 96 ng/mL, *p* = 0.038), which, given the described increase in VEGF-A and pathological capillary proliferation, indicates unique dysfunctions at the interface between the vascular system and adipose tissue.

Inflammatory markers complement this profile: it was demonstrated [77] significantly higher concentrations of IL-11, IL-28A, and IL-29 in the serum of patients, reflecting the formation of a specific immune niche in the subcutaneous tissue; in turn, highly sensitive proteomic profiling [83] revealed the upregulation of 21 circulating inflammatory proteins (including TNFSF14, CASP8, EN-RAGE, MCP-1, IL-8, CCL3, CCL4, MCP-2 to MCP-4, and VEGFA) involved in the biological chemotaxis and migration of immune cells, supporting a strong systemic pro-inflammatory signature in lipedema that exceeds the well-established low-grade inflammation seen in obesity. These findings can be integrated into a coherent metabolic–inflammatory–vascular mechanism: reduced adiponectin and elevated AFABP reflect adipose tissue dysfunction; decreased endostatin alongside increased VEGF-A indicates pathological angiogenesis and vascular permeability; and elevated IL-11, IL-28A, and IL-29, together with upregulation of 21 circulating inflammatory proteins, support a systemic pro-inflammatory signature that exceeds obesity-associated low-grade inflammation. This integrated profile suggests that lipedema involves concurrent dysregulation across metabolic, vascular, and immune axes rather than isolated pathway activation. At the same time, several classic inflammatory markers, such as IL-6, IL-18 and lipocalin-2 (LCN2), have not been consistently elevated in all cohorts [52], and calprotectin (S100A8/A9) data remain limited [11]. However, recent studies report raised CRP in advanced disease stages [61] or CRP and NLR values comparable to obesity-matched controls [98,99]. These findings suggest that a classic acute-phase inflammatory process may not drive lipedema pathogenesis; instead, a distinct chronic pro-inflammatory state is present and becomes more pronounced with disease progression. At the cellular level, in vitro studies confirm this dysfunction: differentiated adipose-derived stem cells (ASCs) from patients with lipedema secrete significantly less adiponectin (consistently) and leptin (results are sometimes inconsistent) into the culture supernatant than cells from healthy individuals. Other single-cohort serum signatures with similar sample sizes have been reported, including pyruvate metabolomics [100] and IgG profiling [101], underscoring the need for validation in larger, independent cohorts. Section 6 discusses the methodological limitations of current biomarker studies, including small sample sizes, cross-sectional designs, obesity/BMI confounding, hormonal and menstrual-cycle effects, disease-stage imbalance, lack of external validation, and differences between superficial and deep adipose tissue sampling.

In light of these limitations, the proposed biomarker panels should be regarded as promising research candidates requiring longitudinal, multicentre validation and standardised clinical phenotyping before any consideration of clinical implementation. Until such evidence is available, biomarker measurements cannot replace clinical evaluation based on established diagnostic criteria.

Integrated biomarker panels spanning the metabolic, inflammatory, and vascular axes are a promising approach toward a more objective diagnosis of lipedema, although they cannot currently replace clinical evaluation.

## 6. Methodological Limitations

However, several significant methodological and clinical limitations hamper current research on the pathogenesis and diagnosis of lipedema and must be considered when interpreting the results. First, observational and cross-sectional studies predominate, which prevents unambiguous conclusions about cause-and-effect relationships between the identified biomarkers and disease development and progression.

Furthermore, these analyses are typically based on relatively small patient cohorts, which, among other things, stems from ethical barriers to obtaining biopsy tissue exclusively from individuals undergoing elective surgery. The limited sample size makes it difficult to generalise the results and increases data variability. The newly developed predictive models and proposed biomarker signatures also lack external validation in independent, multicenter cohorts, which is essential for reliably assessing their clinical utility and stability.

Another key challenge remains the subjectivity of the diagnostic process itself; without approved, objective biological markers, patient enrolment in studies continues to rely on clinical examination and medical history, which carries the risk of classification errors and makes it difficult to compare results across centres. To reduce misclassification at enrolment, several objective tools have been proposed. DXA leg-to-total fat ratios can quantify the disproportionate fat distribution characteristic of lipedema [95]. Ultrasound thickness criteria (e.g., pre-tibial > 11.7 mm, anterior thigh > 17.9 mm) provide reproducible cut-offs for distinguishing lipedema from non-lipedema tissue [94]. Validated screening questionnaires, such as the Lipedema Symptom Screener, offer standardised patient-reported criteria [96]. Researchers have also proposed a case definition to harmonise participant selection across studies [97]. Incorporating these tools into study protocols may reduce classification errors and improve comparability of results across centres.

Furthermore, studies often lack adequate representation of specific disease stages—a particular challenge is recruiting patients in the early (stage 1) and very advanced (stage 3) phases, especially in SAT of the thigh (affected depot). Combining patients at different stages into a single study group to increase statistical power may, in turn, mask the specific mechanisms responsible for disease initiation in the early stage. Coexisting obesity is a significant confounding factor, especially in the later stages of lipedema. The traditional reliance on BMI, which does not capture the disproportionate distribution of adipose tissue in patients with lipedema, makes it difficult to precisely distinguish pathophysiological changes specific to lipedema from those of simple obesity.

In addition, many studies did not control for or standardise the patients’ hormonal status, including the phase of the menstrual cycle at the time of blood collection and their precise premenopausal or postmenopausal status, which—in the case of a condition with a strong estrogenic component—can significantly distort adipokine and lipidomic profiles. At the laboratory and technical levels, tissue collection methods also pose limitations. For example, in advanced multilevel (multi-omics) analyses, transcriptomic and proteomic studies were often conducted on different, only partially overlapping patient subgroups due to difficulties in obtaining sufficient material.

Additionally, tissue routinely collected during liposuction typically consists of a mixture of superficial and deep SAT, which differ in adipocyte size, inflammatory gene expression, and fibrosis; however, separating them precisely during surgery through small incisions is technically infeasible.

## 7. Conclusions

This narrative review synthesises evidence from approximately 102 articles addressing adipose tissue remodelling and adipokine dysregulation in lipedema. The literature indicates that lipedema is characterised by coordinated alterations in subcutaneous adipose tissue, including lipedema as a distinct pathology of SAT, whose development depends on specific, multifaceted changes in the tissue microenvironment. The pathogenesis of this disease is closely linked to local processes, such as progressive adipocyte hypertrophy and hyperplasia, local tissue hypoxia, microcirculatory and lymphatic vessel dysfunction, and changes in stromal and immune compartments, as well as increased extracellular matrix remodelling and fibrosis, in which the deposition of type VI collagen (primarily in the thigh SAT) plays a key role. The immune profile of adipose tissue is frequently described as enriched in CD163+ and CD206+ macrophages; however, these findings should not be interpreted solely within the traditional M1/M2 polarisation framework.

Diagnostic evidence indicates that individual circulating adipokines possess limited clinical utility. Leptin and adipocyte-type fatty acid-binding protein are strongly influenced by total adipose tissue mass and body mass index, whereas adiponectin, fibroblast growth factor 21, chemerin, afamin, and growth and differentiation factor 15 may contribute to multivariable classification models. Additional findings, such as reduced tissue adipsin expression and altered endostatin levels, along with increased interleukin 11, interleukin 28A, and interleukin 29, suggest that lipedema may involve concurrent dysregulation of metabolic, vascular, and inflammatory pathways.

Currently available biomarker models should be regarded as exploratory. Supporting evidence largely comes from small, cross-sectional cohorts, and studies often differ in diagnostic criteria, disease-stage distribution, body mass index matching, hormonal status, tissue sampling, and laboratory methodology. Although some multicomponent models have demonstrated promising discriminatory performance, with accuracy reaching up to 85% in a single cohort, these results lack sufficient external validation. Consequently, biomarker panels cannot presently replace clinical assessment.

Future studies should recruit larger, independently validated multicentre cohorts and use standardised diagnostic criteria, detailed phenotyping, appropriate obesity- and body-composition-matched controls, and harmonised control of hormonal status. Separate evaluation of superficial and deep subcutaneous adipose tissue, longitudinal study designs, and integration of molecular, imaging, and clinical data will be essential for determining whether the proposed biomarkers can support reliable diagnosis and improve understanding of lipedema pathogenesis.

## Figures and Tables

**Table 1:** Clinical staging of lipedema based on morphological features.

Stage	Skin Appearance	Palpation Findings	Nodules	Additional Features
1	Smooth	Thickened hypodermis, soft, pebble-like consistency	Pearl-sized	Early connective tissue loosening and fibrosis
1.5	Early indentations (upper or lower thigh)	Transitional	Pearl-sized, grainy	Intermediate between smooth and textured skin
2	Uneven, dimpling, depressions	Thickened, contracted perilobular fascia, “mattress pattern”	Pearl- to walnut-sized	Fibrotic excess tissue
2.5	Indentations throughout thigh	Early lobule formation	Pearl- to walnut-sized or larger	Overhanging lobules at hip and knee
3	Thinner, less elastic skin	Massive redundant folds	Large subdermal nodules	Overhanging lobules; may impair lymphatic/venous outflow
4 (lipolymphedema)	Variable	Secondary lymphedema	Large, pendulous fat outpouchings	Chronic lymphatic dysfunction; swelling extends to feet/hands

**Table 2:** Primary studies and systemic/tissue profiles of selected adipokines and biomarkers in lipedema. Note: SAT = Subcutaneous Adipose Tissue; SVF = Stromal Vascular Fraction; EC = Endothelial Cells; PC = Pericytes; ASCs = Adipose-Derived Stem Cells; ASCs-T = Thigh ASCs; VIP = Variable Importance in Projection; PLS-DA = Partial Least Squares Discriminant Analysis.

Adipokine	Cohort Size (*n*) & Matching Status	Stage and Sample Depot	Assay Type	Direction of Effect (vs. Obesity/Control)	VIP Score/ Predictor Status	Pathophysiological Role & Methodological Notes
**Leptin**	Felmerer: *n* = 12 vs. 12 (BMI-matched) Nankam: *n* = 20 vs. 20 (BMI-matched Straub: *n* = 76 Kruppa: *n* = 31	Stages 1–3 Serum and thigh SAT	ELISA/Multiplex RT-qPCR (tissue)	**Serum:** No significant differences or elevated in Stage 1 with impaired BMI correlation **Tissue mRNA:** Unchanged or significantly reduced in Stages 2–3 **In vitro:** Increased mRNA in ASCs-T, but lower protein secretion	Low as a single marker (driven by total fat mass)	Regulates satiety and vascular homeostasis. Discrepancies exist between local mRNA transcription and protein secretion into culture supernatant.
**Adiponectin**	Nankam: *n* = 20 vs. 20 (BMI-matched) Mühlberg: *n* = 79 (4 cohorts) Felmerer: *n* = 12 vs. 12 (BMI-matched)	Stages 1–3 Serum and thigh SAT	ELISA/Proteomics RT-qPCR	**Serum:** Significantly higher vs. simple obesity **Tissue mRNA:** Unchanged in SAT and 3D models **In vitro:** Upward trend in gene expression	VIP > 1; Key component of 85% accuracy decision-tree model	Marker of metabolically healthier SAT expansion. Weaker negative correlation with BMI in Stage 1
**FGF21**	Mühlberg: *n* = 79 Nankam: *n* = 20 vs. 20 (BMI-matched)	Stages 1–3 Serum/Plasma	ELISA/Proteomics	**Serum**: Significantly lower in lipedema vs. obesity without lipedema (Mühlberg); no difference in Nankam	Independent Predictor (79–89.7% lower odds per 1 SD increase)	Metabolic regulator; lower levels reflect lower metabolic and inflammatory stress in SAT. Diagnostic performance depends on control group selection.
**Chemerin**	Mühlberg: *n* = 79 Kempa: *n* = 23 premenopausal vs. 23 BMI-matched	Stages 1–3 Serum	ELISA	**Serum**: Significantly lower vs. simple obesity (Mühlberg); unchanged vs. matched obesity in Kempa	VIP > 1; Independent Predictor (56–66.5% lower odds per 1 SD)	Regulates adipogenesis, angiogenesis, and immune cell recruitment. Shows an abnormal loss of physiological correlation with endostatin in lipedema
**AFABP/FABP4**	Mühlberg: *n* = 79 Strohmeier: *n* = 12 vs. 128 Kruppa: *n* = 31	Stages 1–3 Serum, SAT, SVF, EC, PC, ASCs	ELISA RT-qPCR	**Serum:** Lower in lipedema vs. simple obesity **Tissue mRNA:** Stable/unchanged across SAT, SVF, EC, PC, and ASCs	VIP > 1 in PLS-DA, but not an independent predictor in logistic regression	Transports fatty acids. Circulating serum levels reflect total body fat mass, whereas local gene expression in vascular niches remains stable throughout pathogenesis.
**Afamin**	Mühlberg: *n* = 79 (4 cohorts)	Stages 1–3 Serum	Multiplex Immunoassay	**Serum**: No significant univariate difference (*p* = 0.075; 63.4 mg/L in lipedema vs. 75.2 mg/L in obesity)	VIP > 1 in PLS-DA	Contributes significantly to multivariate classification signatures despite the absence of a significant 1-way ANOVA difference
**GDF15**	Mühlberg: *n* = 79 (4 cohorts)	Stages 1–3 Serum	Multiplex Immunoassay	**Serum**: No significant univariate difference (*p* = 0.250; 447.6 ng/L in lipedema vs. 535.4 ng/L in obesity)	VIP > 1 in PLS-DA	High discriminatory value in PLS-DA models alongside metabolic adipokines
**Progranulin (PGRN)**	Mühlberg: *n* = 79 (4 cohorts)	Stages 1–3 Serum	Multiplex Immunoassay	**Serum:** No significant difference (*p* = 0.091; 35.9 µg/L vs. 39.0 µg/L)	VIP < 1 (No discriminatory value)	Systemic levels do not reflect lipedema pathomechanisms; excluded from the diagnostic signature.
**Resistin (RETN)**	Ünlü: *n* = 19 lipedema (BMI 34.4) vs. 15 healthy controls (BMI 25.5); Unmatched	Thigh and Calf SAT Serum	ELISA	**Serum:** No significant difference (*p* = 0.84; 304.1 vs. 392.0 pg/mL)	Low/Non-predictive	Shows no correlation with ultrasound-measured skin or SAT thickness. The marked BMI mismatch between groups limits direct comparisons
**Visfatin (VISF)**	Ünlü: *n* = 19 vs. 15 controls; Unmatched	Thigh & Calf SAT Serum	ELISA	**Serum:** No significant difference (*p* = 0.78; 0.16 vs. 0.16 ng/mL)	Low/Non-predictive	Circulating concentrations are nearly identical between lipedema and control groups.
**Endostatin**	Kempa: *n* = 23 premenopausal lipedema vs. 23 BMI-matched controls	Stages 1–3 Serum	ELISA	**Serum:** Significantly reduced (85 vs. 96 ng/mL, *p* = 0.038)	Vascular Predictor	Potent angiogenesis inhibitor; indicates altered vascular homeostasis and impaired anti-angiogenic signalling at the adipose-vascular interface.
**IL-11**	Wolf: *n* = 5 lipedema vs. 4 controls (BMI-matched)	Stages 1–3 Serum and SVF	Multiplex Assay	**Serum:** Significantly elevated (2.64 ± 0.65 vs. 2.07 ± 0.51 pg/mL)	Promising Inflammatory Marker	Produced primarily by SVF cells; regulates ADSC proliferation and adipogenesis, shaping a specific immune niche.
**IL-28A and IL-29**	Wolf: *n* = 5 lipedema vs. 4 controls (BMI-matched)	Stages 1–3 Serum	Multiplex Assay	**Serum:** Significantly elevated	Immune Niche Component	Reflects a distinct, localised chronic pro-inflammatory niche in subcutaneous tissue.
**Adipsin (CFD)**	Felmerer: *n* = 12 vs. 12 (BMI-matched) Straub: *n* = 76	Stages 1–3 Thigh SAT	RT-qPCR Proteomics	**Serum:** Unchanged in proteomic studies **Tissue mRNA:** Significantly reduced (~1.8-fold) in SAT	Tissue Biomarker	Component of the alternative complement pathway; local transcriptional silencing in SAT may drive abnormal adipocyte expansion
**LCN2 (Lipocalin-2)**	Felmerer: *n* = 12 vs. 12 (BMI-matched) Mühlberg: *n* = 79 (4 cohorts)	Stages 1–3 Serum	ELISA	**Serum:** No significant difference (*p* = 0.558)	No Discriminatory Value	A classic marker of metabolic inflammation; stable levels suggest that lipedema does not exhibit classic insulin-resistance-driven systemic inflammation.

## Data Availability

No new data were created or analyzed in this study. Data sharing is not applicable to this article.

## Abbreviations

The following abbreviations are used in this manuscript:

AT	Adipose tissue
SAT	Subcutaneous adipose tissue
BMI	Body mass index
ECM	Extracellular matrix
SVF	Stromal vascular fraction
sEVs	Small extracellular vesicles
ASCs-T	Thigh adipose-derived stem cells
ASC	Adipose-derived stem cell
VE-cadherin	Vascular endothelial cadherin
CDH5	Cadherin 5
ZO-1	Zonula occludens-1
ANGPTL2	Angiopoietin-like protein 2
OCR	Oxygen consumption rate
CFD	Complement Factor D (Adipsin)
LCN2	Lipocalin-2
GDF15	Growth and differentiation factor 15
PGRN	Progranulin
RETN	Resistin
VISF	Visfatin
AFABP	Adipocyte-type fatty acid-binding protein
FABP4	Fatty acid-binding protein 4
FGF21	Fibroblast growth factor 21
IL-6	Interleukin 6
IL-11	Interleukin 11
IL-18	Interleukin 18
IL-28A	Interleukin 28A
IL-29	Interleukin 29
VEGFA	Vascular endothelial growth factor A
TNFSF14	Tumour necrosis factor superfamily member 14
CASP8	Caspase 8
EN-RAGE	Extracellular newly identified receptor for advanced glycation end products binding protein
MCP-1	Monocyte chemoattractant protein-1
PLS-DA	Partial least squares discriminant analysis
VIP	Variable importance in projection
HbA1c	Glycated haemoglobin
hsCRP	High-sensitivity C-reactive protein
